# Should the Radiologist Always Request a Blood Test Before an Emergency CT Scan in Children

**DOI:** 10.5334/jbsr.3271

**Published:** 2024-01-27

**Authors:** Thomas Saliba, Gervais Kogni Fokou, Paolo Simoni

**Affiliations:** 1 Radiology department, Hopital Universitaire Des Enfants Reine Fablola, 1020 Bruxelles, Belgium; 2 Radiology department, Hopital Universitaire Des Enfants Reine Fablola, 1020 Bruxelles, Belgium; 3 Radiology department, Hopital Universitaire Des Enfants Reine Fablola, 1020 Bruxelles, Belgium

**Keywords:** Child, Kidney, Renal Insufficiency, Hematologic Tests, Emergency Service

## Abstract

Renal failure is relatively common in children presenting to the emergency department, suggesting that the assumption of normal renal function is not always valid. Although some computed tomography (CT) scans necessitate the use of intravenous contrast, one should probably consider whether a blood test is necessary to assess the patient’s renal function and possibly consider other imaging modalities before proceeding. With no pediatric-specific guidelines and no validated pediatric prevention strategies, further research is needed to establish clear recommendations for contrast-enhanced exams in stable and unstable pediatric patients with unknown renal function.

## Introduction

When a child presents to the emergency room suffering from a condition that requires a contrast-enhanced computed tomography (CT) scan, we generally assume that they are not suffering from renal failure. However, some studies show that renal failure is relatively common in children presenting to the emergency department, and thus we must ask ourselves whether or not it is necessary to wait for the results of a blood test in order to verify the glomerular filtration rate of the patient. Currently, there is little literature discussing this subject, with most studies being done on adult populations, with no consensus as to how to best approach the problem [1]. Furthermore, there is even discord among practitioners regarding how to establish a patient’s renal status, with some using questionnaires or putting the responsibility on the referring clinician to check beforehand, though most will still rely on a blood test if there is any doubt [2]. This problem is compounded by the fact that there is currently no consensus as to what constitutes acute kidney injury (AKI) in pediatric patients, though the AKI Network has attempted to standardize the diagnosis [3].

## Discussion

In certain pediatric emergencies, we are sometimes in need of a CT-scan, particularly in cases of trauma or abdominal emergencies. When performing such a scan, it is generally accepted that the benefits of using intravenous contrast outweigh the risks, especially given the sunk cost of radiation exposure for the child, while noting that additional contrast-enhanced images increase the radiation dose. However, given the time pressure, a blood sample with which to judge the state of the patient’s kidneys is seldom performed in pediatrics. Furthermore, although there are risk factors that should prompt a glomerular filtration rate measurement, for example, a history of acute or chronic kidney disease, these may be unknown at the time of presentation [4]. In addition, we have a cognitive bias because most of the other contraindications found in adults are not present in children. One particularly important example is in the case of polytrauma patients, in which a contrast-enhanced study is necessary to visualize vascular and organ damage appropriately. However, studies have shown that up to 30% of children with trauma of various types arrive at the emergency department with AKI, with a meta-analysis finding that the average was around 9%, with an associated fivefold increase in mortality [1]. Furthermore, an American study found 10% of all children admitted to their emergency department whose creatinine was measured at admission and who then went on to be hospitalized either had AKI on admission or developed it within 48 hours [2]. A Nigerian study found AKI in 33% of their emergency room patients, excluding those on dialysis [3]. This obviously raises concerns regarding the assumption that all patients have perfect kidney function and can therefore undergo a contrast-enhanced injection before the results of a blood test.

Other studies, conducted on adults, suggest that the risks of contrast-induced nephropathy may be overblown [3]. Gilligan et al. conducted a study on pediatric patients and found that the risk of AKI was similar in children having undergone contrast-enhanced CT scans and children who had undergone imagery without contrast [3, 8]. They found that AKI was associated with a low baseline glomerular filtration rate, a high body mass index, acquired kidney disease, and nephrotoxic antibiotic exposure, but not with iodinated contrast [3, 8]. This study is reassuring with regards to the possibility of iodinated contrast-enhanced exams inducing AKI in children without pre-existing kidney impairment, though they emphasized that they were not able to draw strong conclusions regarding AKI induction in children with pre-existing kidney impairment [8]. However, Calle-Toro et al. found that the incidence of AKI in children with an estimated glomerular filtration rate of below 60 mL/min/1.73 m2 before a CT scan was higher than in those underwent a contrast-enhanced CT than those who underwent a non-contrast exam [9]. Cantais et al. examined the incidence of contrast-induced AKI in a retrospective study of their patients, finding an incidence of 10.3% [10]. They did not demonstrate an increased 30-day risk of mortality in patients suffering from contrast-induced AKI, though they believed it may be due to a lack of statistical power owing to a small sample [10]. MacDonald et al. also conducted a study on patients having received contrast-enhanced CT scans versus non-enhanced CT scans, finding that the incidence of AKI was slightly higher in patients having undergone non-contrast CT scans, though they theorized it may have been due to selection bias steering away at-risk patients [11]. They found no other significant differences between the groups [11].

Nevertheless, one must keep in mind that, in cases where an urgent contrast-enhanced exam is necessary, it must be performed regardless of the consequences [4]. However, in cases where the patient is stable and we have enough time to wait for the results of the blood test, it is likely wise to wait for the kidney function to be able to suggest alternatives if the patient is found to have AKI.

It may be particularly important to bring attention to this as the patients most in need of contrast-enhanced exams, such as those having suffered trauma, may be those most at risk of AKI.

Although Kidney Disease Improving Global Outcomes (KDIGO) guidelines, which aim to develop and implement evidence-based clinical practice guidelines in kidney disease, exist, it is unclear if these also apply to pediatric patients. In addition, no prevention strategies have been established, with contrast-induced AKI in children being particularly lacking in data, with most results being derived from adult studies [1, 4, 12, 13]. Therefore, because we cannot simply assume that patients have normal kidney function due to their young age, we believe that it is necessary to establish guidelines or a consensus statement specific to pediatric patients to ease the decision of using contrast agents before performing a contrast-enhanced CT in children with stable clinical parameters.

Radiological societies have also produced some guidelines. The European Society of Urogenital Radiology, for example, states that safety considerations in children are similar but not identical to those of adults, with adjustments needed for weight and age, but makes no specific mention of the consideration in cases of emergency or possible renal failure in children [14]. The American College of Radiology states in their 2023 guidelines that renal function measurement is difficult in children and that even normal serum creatinine concentrations cannot exclude renal failure but recommend the use of the Bedside Schwartz to calculate the eGFR [15]. However, as no pediatric-specific measures have been published regarding avoiding contrast-induced nephrotoxicity in children with impaired renal function, they recommend considering the same practices as with adults if one must use iodinated contrast media, while avoiding its use if possible [15].

Recently, the Royal College of Emergency Medicine and the Royal College of Radiologists have weighed-in specifically on the issue of iodinated contrast use in emergency settings and where the induction of contrast-induced nephropathy is a possibility [16]. They recommend its use, regardless of the risk of nephropathy, if the need outweighs the risk [16]. However, while they say that age is not an independent risk factor for contrast-induced nephropathy, they do not specify whether their statement is only valid for adults or is also applicable to children [16].

## Conclusion

In conclusion, we believe that there is a need for caution when considering using intravenous contrast agents in pediatric patients whose renal function is unknown as several studies have demonstrated it can be altered, especially in cases involving trauma.

Although statements from the American College of Radiology, the Royal College of Emergency Medicine and the Royal College of Radiologists, the European Society of Urogenital Radiology, and the National Kidney Foundation dating from 2020 exist, they note that their advice regarding pediatrics is largely derived from adult studies and thus needs further research. Current studies are reassuring with regard to contrast-injection in children without pre-existing renal impairment. However, evidence is conflicting regarding the effects on children with pre-existing renal impairment, as some studies showing an increased risk while others do not. This emphasizes the importance of considering the risk of inducing AKI or injecting contrast into a child already suffering from renal failure. As there are currently no established pediatric guidelines, we believe that this area needs further study in order to provide a clear path to follow when dealing with children when time is limited and one must quickly weigh the risks of a contrast-enhanced exam against the benefits.
